# Choosing and Using a Plant DNA Barcode

**DOI:** 10.1371/journal.pone.0019254

**Published:** 2011-05-26

**Authors:** Peter M. Hollingsworth, Sean W. Graham, Damon P. Little

**Affiliations:** 1 Genetics and Conservation Section, Royal Botanic Garden Edinburgh, Edinburgh, United Kingdom; 2 University of British Columbia Botanical Garden and Centre for Plant Research, Faculty of Land and Food Systems, and Department of Botany, University of British Columbia, Vancouver, British Columbia, Canada; 3 Cullman Program for Molecular Systematics, The New York Botanical Garden, Bronx, New York, United States of America; Biodiversity Insitute of Ontario - University of Guelph, Canada

## Abstract

The main aim of DNA barcoding is to establish a shared community resource of DNA sequences that can be used for organismal identification and taxonomic clarification. This approach was successfully pioneered in animals using a portion of the *cytochrome oxidase 1* (*CO1*) mitochondrial gene. In plants, establishing a standardized DNA barcoding system has been more challenging. In this paper, we review the process of selecting and refining a plant barcode; evaluate the factors which influence the discriminatory power of the approach; describe some early applications of plant barcoding and summarise major emerging projects; and outline tool development that will be necessary for plant DNA barcoding to advance.

## 1. Selecting (and Refining) a Plant Barcode

### 1.1. Selecting a core-barcode

Three important principles of DNA barcoding are standardisation, minimalism, and scalability. Translating this into the selection of barcoding regions involves choosing one or a few standard loci that can be sequenced routinely and reliably in very large and diverse sample sets, resulting in easily comparable data which enable species to be distinguished from one another. The standard animal *CO1* DNA barcode fits these criteria well [1]. It is a haploid, uniparentally-inherited, single locus that shows high levels of discriminatory power [2]. It is a protein-coding region present in high-copy numbers per cell, and in animals it is not prone to drastic length variation, strong secondary structure, microinversions, or frequent mononucleotide repeats. These characteristics, combined with well-developed primer sets, result in the routine recovery of high quality sequences from many animal clades and facilitate sequence recovery from poorly-preserved samples. *CO1* sequences can be consistently orientated, aligned with little supervision, and be translated to diagnose pseudogenes and identify sequencing errors.

Finding a plant equivalent has proved difficult. The generally low rate of nucleotide substitution in plant mitochondrial genomes precludes the use of *CO1* as a universal plant barcode [3]. Instead, the search for a plant barcode has involved looking outwith the mitochondrial genome and from the outset many researchers have accepted that multiple markers will be required to obtain adequate species discrimination.

**Figure 1:**
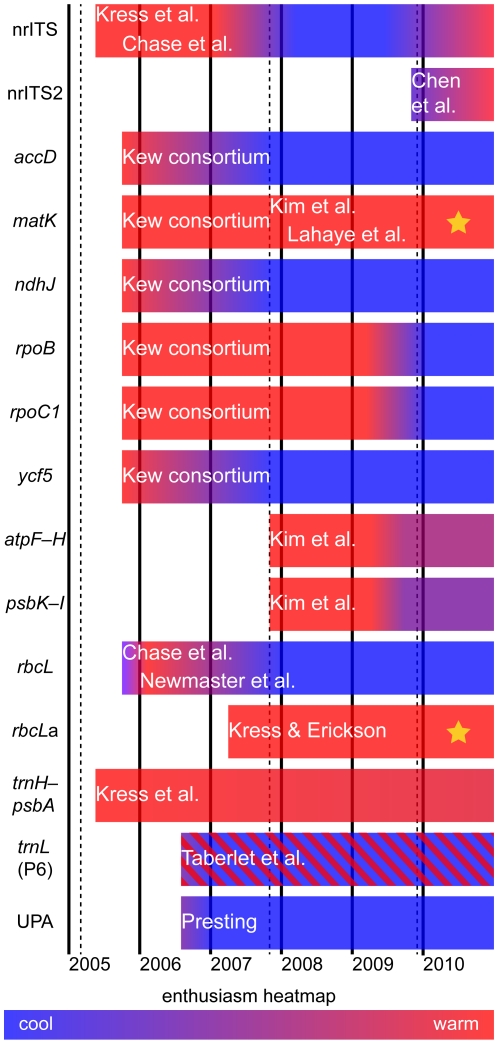
Schematic timeline of the consideration of different markers as plant barcodes. Colours (red = warm; blue = cool) represent an informal measure of enthusiasm among DNA barcoding researchers in the systematics community for CBOL and iBOL adoption of different markers. The different shading of *trnL* (P6) reflects the parallel use of the P6 loop for DNA profiling of degraded DNAs in ecological studies (see text). * = the two markers that form the core-barcode for land plants. *rbcL* a is used in this figure to distinguish this shorter barcode region of the gene proposed by Kress and Erikson [7] and the full length (ca. 1400 bp) gene sequence of *rbcL*. Elsewhere in the text, when we refer to *rbcL* we are referring to the short barcode region. The dashed lines indicate the timing of three international barcoding conferences in London (2005), Taipei (2007) and Mexico City (2009). The consideration of the different markers as barcodes are from the following sources: Kress et al. [5], Chase et al. [102], Chen et al. [54], Kew consortium [4], [6], Kim et al. see [103], Lahaye et al. [8], Newmaster et al. [104], Kress and Erickson [7], Taberlet et al. [44], Presting et al. [105].

An historical overview of the search for a plant barcode is summarized in Figure 1, and discussed briefly below. Following initial *in silico* and laboratory-based assessments of the suitability of various coding and non-coding plastid markers (e.g. [4], [5]; Table 1), four main suggestions for a plant barcode were proposed by three different research groups/research consortia from the systematic community. These proposed barcodes involved various combinations of seven plastid markers. These were *rpoC1*+*rpoB*+*matK* or *rpoC1*+*matK*+*trnH-psbA* [6]; *rbcL*+*trnH-psbA* [7] and *atpF-H*+*psbK-I*+*matK* (K. J. Kim et al., unpublished). Various combinations of these markers were discussed at the 2^nd^ International Barcode of Life conference in Taipei, but no agreement was reached. The following year, Lahaye et al. [8] proposed that *matK* alone should constitute the plant barcode.

**Table 1:** Characteristics of different markers that have routinely been included in plant barcoding studies.

Marker	Genomic source	Type	Approx number of GenBank accessions	Approx number of GenBank genera	Approx number of GenBank species	Median amplicon length (bases) in completely sequenced plastid genomes	IQR amplicon length (bases)	Amplicon length range (bases)	Number of samples used to estimate amplicon length	Freq. of amplicons with mono-nculeotide repeats ≥10 bases
**nrITS**	Nuclear	Transcribed spacers and 5.8S gene	102684	13307	52450	705	683–724	407–1630	5020	0.013
**nrITS2**	Nuclear	Transcribed spacer	111370	15817	57579	494	492–506	157–670	646	0.005
***atpF-H***	Plastid	Inter-genic spacer	1180	274	664	669	578–707	390–918	134	0.440
***matK***	Plastid	Protein coding	34647	7454	22701	889	880–889	862–910	132	0.235
***psbK-I***	Plastid	Inter-genic spacer	1241	208	626	468	444–492	112–1253	134	0.500
***rbcL***	Plastid	Protein coding	27725	8959	20374	654	654–654	654–654	134	0.000
***rpoB***	Plastid	Protein coding	3341	751	1970	548	548–548	536–590	132	0.008
***rpoC1***	Plastid	Protein coding	5314	1110	3075	616	616–616	610–622	132	0.000
***trnH-psbA***	Plastid	Inter-genic spacer	23526	2833	11539	509	401–617	226–934	135	0.296
***trnL-F***	Plastid	Intron and inter-genic spacer	59197	9129	35130	994	907–1037	201–2114	132	0.280
***trnL*** ** (P6)**	Plastid	Intron	70811	10561	38329	87	83–91	51–135	130	0.054

The number of sequences deposited in GenBank for each marker was estimated from sequence annotations and should be considered only as an approximation. Estimates of amplicon size (including priming sites) and frequencies of mononucleotide repeats were made from all annotated land plant whole plastid genomes deposited in GenBank and for nrITS from a curated set of nrITS sequences. Mononucleotide repeats less than 10 bp in length were excluded because these generally do not affect sequence quality [36], [37].

One of the biggest challenges in reaching agreement on a plant barcode was a lack of comparative data encompassing all candidate markers and a broad taxonomic sample. The sequential timing of different proposals (Figure 1) effectively meant that some groups were proposing new markers and primers, as others were completing their projects. Two research groups published direct comparisons of the seven candidate markers and both concluded (a) that several different marker combinations gave equivalent performance, and (b) that none of the proposed barcodes was perfect in every respect [3], [9]. The same conclusion was reached by Seberg and Petersen [10] whose study included *rpoC1*, *matK* and *trnH-psbA*.

Agreement on a common barcode is necessary for plant barcoding to progress towards the creation of a shared community resource. To facilitate and formalise the selection of a plant barcode, the Consortium for the Barcode of Life (CBOL) instigated the formation of a working group with representation from the different research groups/research consortia from the systematics community that had proposed or tested the seven leading candidate barcoding markers. This involved data generation, data pooling and joint analyses of the data, assessing the candidate markers against three criteria (a) universality (ease of amplification and sequencing), (b) sequence quality, and (c) discriminatory power. The outcome of these trials was that although some markers could be eliminated from consideration (e.g. *rpoC1* and *rpoB* showed markedly lower discriminatory power), there was no straightforward solution as to which should form the plant barcode, as each of the candidate markers had different strengths and weaknesses. The majority preference of the CBOL Plant Working group was to recommend a core-barcode consisting of portions of two plastid coding regions, *rbcL*+*matK*, to be supplemented with additional markers as required [11]. The *rbcL* barcode consists of a 599 bp region at the 5′ end of the gene, located at bp 1–599 (including primer sites) in the complete *Arabidopsis thaliana* plastid genome sequence (gi 7525012:54958–56397). The *matK* barcode region consists of a *ca.* 841 bp region at the center of the gene, located between bp 205–1046 (including primer sites) in the complete *A. thaliana* plastid genome sequence (gi 7525012:2056–3570).

The choice of *rbcL+matK* as a core barcode was based on the straightforward recovery of the *rbcL* region and the discriminatory power of the *matK* region. *matK* is one of the most rapidly evolving coding sections of the plastid genome [12], and is perhaps the closest plant analogue to the *CO1* animal barcode. Unfortunately, *matK* can be difficult to PCR amplify using existing primer sets – particularly in non-angiosperms (see below). In contrast, the barcode region of *rbcL* is easy to amplify, sequence, and align in most land plants and provides a useful backbone to the barcode dataset, despite it having only modest discriminatory power. Two-marker plastid barcodes gave better discrimination than single marker barcodes, but no other 2-marker or multi-marker plastid barcode gave appreciably greater species resolution than the *rbcL*+*matK* combination [11]. As both of these markers are coding regions, electronic translation of sequences from DNA to amino acids can be used to automate checks for editing/assembly errors, the presence of psuedogenes, and correct sequence orientation. The coding and hence directly alignable nature of the data also facilitates character based analyses and comparative analyses of DNA barcode diversity among taxonomic groups and geographical regions.

In recommending *rbcL*+*matK* as the core-barcode for land plants, two challenges were clear from the outset. First, further work is required on *matK* primer development to enable routine and efficient PCR and sequencing. Second, the discrimination success of *rbcL*+*matK* in plants is typically lower than *CO1* in animals. These challenges are discussed in turn below.

#### *a.* MatK *Primer development*

As more datasets are published, we can more accurately estimate the extent of primer universality for *matK*. Using the best currently available ‘universal’ primer pair (3F/1R; K. J. Kim unpublished) on diverse sample sets typically results in PCR and sequencing success of ca. 70% in angiosperms. Use of a secondary primer pair (390F/1326R; [13]) can increase amplification and sequencing success by another ca. 10%. This *matK* recovery rate clearly needs improvement for plant barcoding to be cost-effective and efficient. Furthermore, *matK* is not recoverable from some bryophyte and fern groups with available primer sets, most of which were designed for angiosperms. Ferns in particular represent a challenge for *matK* recovery as genome rearrangements mean that the gene is not flanked by conserved *trnK* exons in some clades [14], [15], creating additional difficulties in generating full-length *matK* sequences from which to design primers for the barcode region. Three complementary strands of research are currently being pursued to improve the amplification and sequencing of *matK* from land plants. Firstly, clade-specific primers are being designed to improve recovery within a given taxonomic group (e.g. [16] for angiosperms; [15], [17] for other land plants). Secondly, modifications are being made to existing ‘universal’ primers and reaction conditions in an attempt to increase their success rate, including ‘mix-and-match’ of individual primers among existing primer pairs. Thirdly, work is underway to design primer cocktails around existing *matK* barcode priming sites. A project funded by the Gordon and Betty Moore foundation addressing these issues is scheduled for completion in late 2011.

#### *b. Discrimination success*

Species discrimination with plant barcodes is typically lower than with *CO1* in animals. Obtaining precise figures is difficult as most studies to-date have focused on assessing the *relative* rather than *absolute* discriminatory power of different barcoding regions. Levels of discrimination vary greatly among taxa and study designs, but species discrimination figures less than 70% in plants are not uncommon (Table S1 list discrimination success from 42 studies; discussed in detail later in the paper). In these situations, where the barcode does not provide a unique species identification, it instead identifies to ‘species group’ (typically a local group of closely related congeners). Additional studies with greater sample density are required to establish the situations in which the *rbcL*+*matK* barcode provides ‘species group’ versus unique species identifiers.

Given these two joint challenges (*matK* primers needing improving, and uncertainty as to the absolute levels of discriminatory power of *rbcL*+*matK*), the designation of *rbcL*+*matK* as the standard core-barcode for land plants by CBOL is subject to a review of its performance scheduled for late 2011. This ‘review period’ was adopted to enable plant barcoding studies to commence in earnest, whilst allowing for modifications to protocols should they be required. During this review phase, continued sequencing and exploration of the properties of other non-coding markers is recommended (particularly *trnH-psbA* and the internal transcribed spacers of nuclear ribosomal DNA nrITS/nrITS2). This is to establish whether it is necessary to formalize the routine incorporation of other markers into the plant barcode (rather than the current *ad hoc* use of supplementary barcodes – see below).

### 1.2. Using supplementary markers/additional barcodes

The selection of *rbcL*+*matK* as a core two-marker barcode was based on the observation that in the available datasets, there was a plateau in discriminatory power such that no universally appreciable gains were seen beyond two plastid markers [11]. Likewise, there was no overall gain in the use of more variable non-coding plastid regions compared to these two coding regions [11]. Thus, based on the data gathered to-date, a limiting factor appears to be the extent to which plastid haplotypes track species boundaries, rather than a shortage of variable characters per se. This observation emerges when discrimination success focuses on situations where multiple individuals have been sampled from multiple species (e.g. where there is some requirement for members of a species to ‘group together’). In cases where species are represented by single individuals, and the success criterion is simply whether these individual samples can be told apart, the use of more markers, and more variable markers, will naturally give the appearance of increasing discrimination success, but in many cases this will be due to autapomorphies or sequencing errors.

This does not mean additional plastid markers never improve discrimination success, rather it means that to-date no other combination of plastid markers has been identified that will routinely distinguish appreciably more species than a *rbcL+matK* barcode. The problem of the idiosyncratic performance of different markers in different taxonomic groups was well summarized by Fazekas et al. [3] who noted “regardless of the region(s) ultimately adopted for plant barcoding, there will always be some species that would be better resolved by some other region”. In this next section, we discuss the range of additional markers that researchers are using in plant barcoding studies that can form useful supplements to the *rbcL+matK* core barcode.

#### *a. Widely used plastid markers*

Beyond the core *rbcL*+*matK* barcode, the most widely used plastid barcoding marker is the intergenic spacer *trnH-psbA*. This region is straightforward to amplify across land plants, and is one of the more variable intergenic spacers in plants [18]. It has been used successfully in a range of barcoding studies (e.g. [19]–[21]) and is an obvious choice of a supplementary barcode. In directly comparable sample sets it has higher species discrimination success than *rbcL*+*matK* in groups such as *Ficus* [22] and *Alnus* [23] and improved resolution in complex groups such as *Quercus* [24] and *Salix* [25]. The presence of duplicated loci can lead to problems in a small number of groups (e.g. *Pinus* [26], [27]; cycads [28]; *Eryngium* [29]). In some conifers and monocots, the region is in excess of 1000 bp [6], [9], whereas in bryophytes it can be less than 100 bp [30]. Microinversions are not uncommon [31], and these may need accounting for (detection/reorientation/removal) in data analyses as homoplastic microinversions can lead to over-estimates of genetic differences between samples and thus to erroneous groupings of unrelated sequences [31], [32], although in some circumstances uncorrected microinversions provide additional characters for species discrimination [33]. One of the main concerns associated with the use of *trnH-psbA* as a standard barcode was the premature termination of sequencing reads by mononucleotide repeats leading to unidirectional reads in up to 30% of sequences (e.g. [11], [34], [35]). However, experimentation with new polymerases has led to improved sequence quality in the presence of mononucleotide runs up to 13 bp [36], [37]. If these protocols routinely work for large sample sets it should lead to an increase of bi-directional sequencing reads for this marker.

The *atpF-atpH* and *psbK-psbI* intergenic spacers were proposed as plant barcoding regions at the second international Barcode of Life Conference (K. J. Kim unpublished). These two markers have not been widely used in plant systematic and phylogeographic studies and as a result there is a paucity of data on their performance. In the study by the CBOL Plant Working Group [11], *psbK-psbI* showed high levels of discriminatory power, but lower sequence quality and universality, whereas *atpF-atpH* showed relatively modest discriminatory power, intermediate sequence quality and universality. Some recent studies have provided positive reports on the performance of both *atpF-atpH* (e.g. [38], [39]) and *psbK-psbI* [40], and they are reported as proving extremely useful in studies on the Korean flora (Ki-Joong Kim, pers. comm.).

The *trnL* intron and the intergenic spacer between *trnL* and *trnF* have been widely used in plant systematics and phylogeography since the early 1990s. This frequent use is attributable to the early publication of a robust set of primers that allow routine recovery [41]. The regions are generally simple to sequence, although mononucleotide repeats (Table 1) can impact on sequencing reads in some taxa. Duplicated copies of the *trnF* gene have been reported in the Brassicaceae [42], [43], and whole plastid genomes show a loss of the intergenic spacer in a few taxa (e.g. *Manihot esculenta* gi 169794052, *Selaginella moellendorffii* gi 255961289) and loss of the intron in others (e.g. *Lathyrus sativus* gi 295136900, *Lotus japonicus* gi 13518417, *Trifolium subterraneum* gi 219673952). Some studies have noted that other regions of the plastid genome may be more variable and informative for plant phylogenetic studies [18], but a major strength of the *trnL* intron for species identification is the presence of a small stem-loop structure within the intron, the P6 loop [44]. P6 has conserved priming sites flanking a variable loop of ca 10–143 bp. This very short ‘minibarcode’ has proved very useful to ecologists studying highly degraded DNAs and using next generation sequencing technologies to assess the diversity of complex environmental samples (e.g. faeces; [45]). This ‘*trnL* approach’ of ecological barcoding has developed somewhat in parallel to the major international barcoding consortia of the *International Barcode of Life Project* (iBOL) and the *Consortium for the Barcode of Life* (CBOL). One contributing factor to this is that the primers for the *trnL* intron P6 loop are the subject of a patent filing (in force in Europe, pending in the USA, Canada and Japan; March 2011). This does not prevent researchers from using the region for non-commercial research, but it does conflict with the working models of the iBOL and CBOL initiatives. These are based on involvement of the international scientific community in developing an open-access shared resource without constraints or patents limiting the use of regions and primer sets.

#### *b. Widely used nuclear regions*

The internal transcribed spacers from nuclear ribosomal DNA (nrITS) are an obvious choice of a supplementary barcode in groups in which direct sequencing is possible [11], [46]. In some parasitic plants with highly reduced plastid genomes (e.g. [47]) it may represent the only viable currently available barcode (although *matK* may be retained in some fully heterotrophic plants; [48]). The (generally) greater discriminatory power of nrITS over plastid regions at low taxonomic levels is well established in plant molecular systematics, and it has been clear from the outset that in groups where nrITS works well, it will be frequently used as a DNA barcode. Several recent studies have shown nrITS discrimination among plant species that shared plastid haplotypes (e.g. [22], [23], [33], [49], [50], see Table S1). However, there are three primary concerns about nrITS that have thus far prevented it from being a core component of the plant barcode. First, incomplete concerted evolution can lead to divergent paralogous copies within individuals [51], [52]. At best, divergent copies require careful and consistent scoring of sites with polymorphic bases (difficult in high-throughput barcoding situations and hard to replicate across laboratories) and at worst, divergent copies can prevent readable sequences from being obtained. In addition, different variants may be obtained from a given sample depending on the amplification strategy, the primers used, and PCR efficiency – resulting in potentially different species assignments based on different laboratory protocols or chance (e.g. [53]). A second concern is that of fungal contamination [51], particularly in cases where plants contain fungal endophytes. Finally, although a number of nrITS primer sets are available, it can be difficult to amplify and sequence this region from diverse sample sets. For example, Gonzalez et al. [19] reported PCR and sequencing success of 41% from a sample of 285 tropical trees.

An alternative to the use of the entire assemblage of ITS1-5.8S-ITS2 is to use just a portion of the region as a barcode, namely nrITS2 [54], [55]. This approach has been useful in several studies (e.g. [56]–[58]) and it has been argued that focusing on the nrITS2 region reduces amplification and sequencing problems associated with the entire nrITS assemblage [54]. Certainly, the generally shorter length of the target region can make routine sequencing easier than entire nrITS, and in general the nrITS2 region is more length-conserved than nrITS1, making it a more predictable amplicon to work with [54]. The use of nrITS2 thus involves a trade-off between using a small portion of the nrITS assemblage to make recovery and sequencing easier, while sacrificing the number of available characters. Further sampling is required to assess the extent to which this use of fewer characters reduces discrimination success of nrITS2 compared to the entire nrITS region (e.g. Liu et al. [59] found a marked decrease in discriminatory power for nrITS2 compared to the combined ITS1-5.8S-ITS2 assemblage).

The extent of problems with the use of nrITS (or nrITS2) such as paralogy, polymorphic sites, and sequence quality must be rigorously quantified. The limitations of the internal-transcribed spacers have been well documented in general terms [51], [52]. However, without a formal empirical estimate of the number of plant groups in which the problems are likely to occur, it is not possible to know whether these are truly pervasive problems that are likely to impact a large proportion of barcoding studies, or if these problems will affect a relatively small number of species/samples relative to the gain in discriminatory power.

#### *c. Alternative sources of markers*

*Complete plastid genomes*: Decreasing costs and increasing power of next generation sequencing technologies are making sequencing of complete plastid genomes relatively straightforward. The (generally) conserved gene order and size of plant plastid genomes make automated data processing and analysis tractable. Obviously, having entire plastome sequences (compared to a few barcoding markers) is no bad thing, and would side-step some of the complexities associated with partially overlapping reference databases that are a result of different research groups using different supplementary plastid barcodes. The use of complete plastome sequences as DNA barcodes, has been suggested by several authors (e.g. [60], [61]). However, the cost of complete plastome sequencing with current technologies still far exceed that of Sanger sequencing a small number of markers. In addition, sample preparation is not always straightforward (e.g. problems with genome recovery in cases with highly rearranged genomes or degraded DNA). Likewise, assembling sequence reads into plastomes in the absence of a reference sequence remains labor intensive, and among closely related species, it will be critical to establish informatics protocols that ensure sequencing errors do not override any subtle-but-real differences among plastomes. Finally, and most importantly, it remains a concern that the critical factor limiting the success of plastid barcodes is not a shortage of variable characters; rather it is the fact that plastid haplotypes frequently do not completely track species boundaries (discussed by Fazekas et al. [2]; see below). Thus while completely sequenced plastid genomes will undoubtedly help in a number of cases, in many others they are likely to show with great precision (and not inconsiderable expense) that the plastid haplotype in question is not a good marker for a given species.

*Low copy/single copy nuclear genes*: Several nuclear regions have been used in plant phylogenetic studies, such as *waxy*, *leafy*, *alcohol dehydrogenase* and *phytochrome* genes [62]. Bioinformatic screens of transcriptome and whole genome sequences have further identified gene regions that tend to be single copy in divergent lineages and hence represent a promising source of markers (e.g. [63]–[65]) and primer sets that aim to routinely amplify single copy sequences across large clades have been developed [64]. As more sequence data become available from initiatives like the 1KP project (1000 transcriptomes from phylogenetically divergent land plants; http://www.onekp.com/index.html) and the 1000 Plants and Animals Genome project (www.ldl.genomics.cn/page/proposeplant.jsp), prospects are improving for efficient routine recovery of nuclear sequence data. However, the challenges involved in obtaining a common set of nuclear markers that can be easily amplified and sequenced in large phylogenetically divergent sample-sets remain non-trivial: primer site mutations, gene duplication, recombination, insertion of transposable elements, polyploidy and heterozygosity all combine to create a set of practical challenges. Coding regions need to be identified that have variable sites suitable for species level differences, or which contain sufficiently conserved intron sizes and positions for the design of exon-primed intron-crossing (EPIC) markers. Nevertheless, progress in this field is important as being able to routinely sequence multiple nuclear loci will ultimately be required to provide species level resolution in the many plant groups where species histories are complex and/or where speciation is recent.

One alternative future to cracking the problem of careful development of set of single copy nuclear sequences, is that next generation sequencing technologies will ultimately get to the point that obtaining vast amounts of sequence data from many individual samples is feasible, which may take care of the problem by permitting the fullest possible description of species boundaries using genetic data. However, although approaches like RAD sequencing are a step in this direction [66], the large size of many plant genomes, and the phylogenetic diversity represented by land plants makes the ‘solve-the-problem-with-masses-of-data’ approach currently computationally impractical and prohibitively expensive with the current pool of technologies. This landscape is, however, changing rapidly. If sequencing costs continue to fall, and critically, if user-friendly and effective bioinformatic pipelines can be established, prospects are improving for harnessing advances in next generation sequencing technologies for DNA barcoding type projects (e.g. [67]).

## 2. Factors Influencing the Discrimination Success of Plant Barcodes

The preceding section discussed discriminatory power in relation to the choice of barcode markers. In this section, we explore which biological factors influence the success of plant barcoding projects. Table S1 lists examples of plant studies that have provided estimates of discriminatory success with DNA barcodes, or from which analogous data could easily be obtained. In general, when the sample set is geographically constrained, levels of discrimination can be high (e.g. [20], [68]) – a result of species in the sample being distantly related. In contrast, as one moves towards dense sampling of individual taxonomic groups, the number of distinct species decreases due to shared barcodes among species (e.g. [9]).

There are a number of features that can potentially contribute towards a lack of unique species identification with DNA barcodes. For DNA barcoding to work successfully, it requires sufficient time since speciation for mutations and/or drift to lead to a set of genetic characters ‘grouping’ conspecific individuals together, separate from other species. In clades where speciation has been very recent, or rates of mutation are very slow, barcode sequences may be shared among related species. Particularly problematic groups include woody species with long generation times and/or slow mutation rates (e.g. *Araucaria*), and also groups which have radiated recently and rapidly (e.g. *Inga* [9]).

Polyploid speciation can lead to incongruence between barcode sequences and taxon concepts [2]. Where multiple allopolyploid species share a common parent species, they may have identical plastid sequences, whereas independent origins of allopolyploid species can lead to taxa treated as conspecifics possessing divergent haplotypes. Species that have originated by allo- or autoploidy will, at least initially, share their plastid haplotype(s) with a diploid progenitor (over evolutionary time-scales this is expected to be less of a problem for DNA barcoding as polyploid derivatives diverge and speciate further).

In taxonomically complex groups (TCGs [69]) where species limits are often very narrowly defined, exact species identifications using a barcoding approach are unlikely. One or a few markers cannot usually resolve the complexity in TCGs resulting from recurrent ecotypic origins of taxa, or where (micro-) species arise through some process like recurrent ploidy transitions (as outlined above), recent hybrid speciation, or apomixis (e.g. *Euphrasia*, *Taraxacum*, *Sorbus*, *Dactylorhiza*; [69]). Even in groups where species limits are relatively clear (i.e. most individuals can readily be assigned to a given species), past hybridization can lead to shared plastid haplotypes among species. ‘Chloroplast capture’ has long been documented in the phylogenetics literature [70], and in various phylogeographic studies on groups like *Packera* [71], *Pinus* [72], *Quercus* [73], and *Salix* [74].

**Figure 2:**
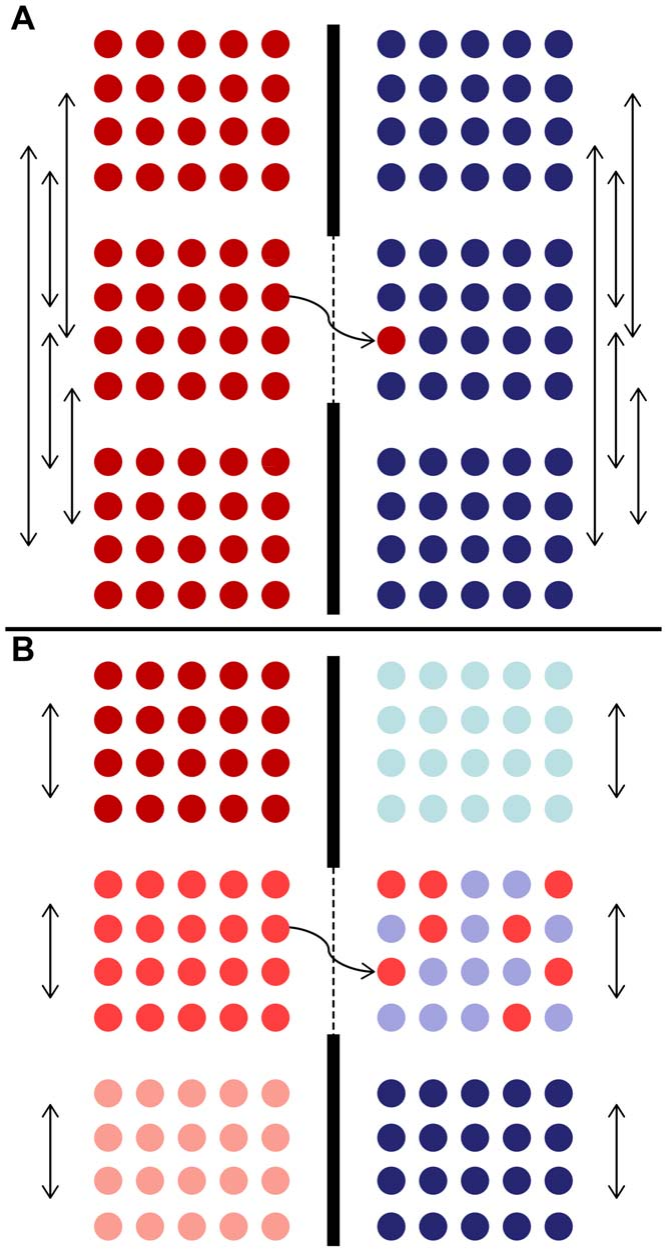
Schematic representation of the impacts of intra-specific gene flow on species discrimination success. Parts (A) and (B) each represent two species (one shades of red, one shades of blue), each consisting of three populations. The black line between the species indicates a barrier to gene flow, with the thickness of the line indicating the strength of the barrier. In (A) intra-specific gene flow among populations is high (indicated by the vertical arrows). Thus, where gene flow occurs between species (wavy arrow), there is a barrier to extensive neutral introgression because establishment of immigrant alleles is prevented by a regular influx of conspecific alleles from other populations. In (B) intra-specific gene flow among populations is low. Thus populations are more differentiated from one another and are less likely to show taxon-specific barcode markers. In addition, the flux preventing establishment of introgressed alleles is lower because it involves only alleles in the (middle) recipient population and not the other populations of the ‘blue’ species.

The situations described above are well-established scenarios which common sense dictates as being likely to have an impact on the levels of species discrimination success of a given barcoding study. A somewhat more subtle but potentially important factor was identified by Currat et al. [75] and Petit and Excoffier [76], namely that dispersal ability may be a predictor of species discrimination success, and that there may be an inverse correlation between intra- and inter-specific gene flow. The logic flow behind these ideas is summarized in Figure 2, and outlined below.

In species where dispersal is poor, populations are relatively isolated from one another. The first consequence of this, is that individual neutral mutational variants can be slow to spread throughout a species’ range, and the time taken for a species to reach ‘monophyly’ for a given locus will be slower than for a species whose populations are connected by regular gene flow [76]. Thus poorly dispersed species may be less likely to show species-specific barcodes in the first place. A secondary consequence of poor dispersal is that the permeability of a species to inter-specific gene flow may be increased [76]. In situations where two species with high levels of intra-specific gene flow co-occur and hybridize, introgression may be restricted due to demographic competition against introgressed alleles from the recurrent influx of intra-specific alleles (Figure 2a). In contrast, where intra-specific gene flow is low, the level of demographic competition against introgressed alleles will be lower (Figure 2b). This is because any introgressed ‘foreign’ alleles are only competing against the alleles in the local population at the site of hybridization, rather than with a wider interconnected network of populations. Thus there may be an increased likelihood of successful inter-specific gene flow for neutral markers in species with poor intra-specific dispersal.

Two studies have provided elegant tests of this hypothesis using conifer systems in which different organelle markers have different dispersal abilities [77], [78]. Thus in *Pinus* and *Picea* (as in many other gymnosperms), mitochondrial DNA variants are maternally inherited, and hence travel only as far as seed is dispersed. In contrast (and unlike most other land plants) plastid markers are paternally inherited and thus also travel in pollen, potentially covering much larger distances. In the studies of Du et al. [77] and Zhou et al. [78], paternally inherited (and better dispersed) plastid markers showed consistently greater congruence with morphological species boundaries, than maternally inherited (and more poorly dispersed) mitochondrial markers.

Unlike conifers, angiosperm plastid DNA is typically maternally inherited [79]. Thus, plastid variants are only dispersed by seed and do not travel as far as nuclear alleles which are dispersed by both pollen and seed [80]–[82]. There are many exceptions to this broad generalization, but for many species pollen dispersal distances are vast compared to relatively local movements of seed. Pollen : seed flow ratios derived from joint nuclear-organelle *F*_ST_ estimates typically show much higher pollen-flow than seed-flow [80]–[82], and measures of population differentiation for maternally inherited markers are typically much higher than for nuclear markers [81].

This has two key consequences for plant barcoding. First, due to their limited dispersal plastid plant barcodes have a built-in limitation to tracking species boundaries in some cases. This may be one of the reasons that plant plastid barcodes show lower discriminatory power than animal barcodes: although some animal species show male-biased dispersal, most terrestrial animals lack a mechanism for such large sex-biased dispersal asymmetries as those seen in plant species that broadcast their pollen over extremely long distances compared to the movement of their seeds.

Secondly, these studies by Currat et al. [75] and Petit and Excoffier [76] provide a satisfactory explanation for what has otherwise been a rather puzzling result to explain – why chloroplast capture in plants appears to be so frequent in the absence of obvious nuclear introgression. Thus there are numerous situations in which multiple species share plastid DNA haplotypes, yet remain distinct for nuclear markers like nrITS [49], [83]. Although various selective arguments may explain this phenomenon, it may simply reflect the difference in the dispersal abilities of these marker systems – plastid DNA is often more poorly dispersed than nrITS. Taken together, these results provide an additional impetus to explore options for routinely augmenting plastid barcodes with nuclear markers.

In terms of translating the above set of observations into predictions of which groups will be likely to show high levels of discrimination success, Table 2 provides a non-quantitative approximation of species attributes which are likely to influence the likely outcomes of a study. Thus groups that are taxonomically oversplit, those in which hybridization and/or polyploidy is frequent, those that have radiated recently, those that have slow mutation rates, and those with limited seed dispersal are predicted to show lower discrimination success with DNA barcodes. More studies are required to turn these generalizations into quantitative predictors.

**Table 2:** Key factors likely to lead to lower levels of success in species discrimination in DNA barcoding studies.

Factor	Situations where lower species discrimination success is expected
Hybridization	Groups in which hybridization is frequent and hybrids show some fertility
Polyploidy	Groups in which speciation frequently involves polyploidy
Life history	Groups of long lived organisms and/or those with slow mutation rates
Breeding system	Species groups consisting of closely related agamospermous or autogamous lineages
Species history	Species groups where speciation has been recent and rapid, or where continuously large historical population sizes lead to maintenance of ancestral polymorphism
Level of taxonomic ‘splitting’	Groups in which the species limits have been very narrowly defined
Seed dispersal	Angiosperm species groups in which seed dispersal is poor (plastid barcodes)

## 3. Applications of DNA Barcoding in Plants

Plant DNA barcoding research is shifting beyond performance comparisons of different DNA regions towards practical applications. These applications can be split into two broad categories. One is to provide insights into species-level taxonomy and contribute towards the taxonomic process of defining and delimiting species. The second, and major application, is to assist in the process of identifying unknown specimens to known species.

DNA barcoding in plants is most likely to provide insights into species-level taxonomy in groups with simple morphologies, those with very broad distributions, those that are diminutive in size, and/or those that have received inadequate taxonomic attention to adequately characterize the diversity they contain (e.g. situations where morphology-based taxonomy is challenging, or has not been done thoroughly). One plant group in which DNA barcoding approaches are providing useful insights into cryptic species diversity is bryophytes ([84], [85] Hollingsworth et al. unpublished). Bryophyte species in general lack many of the ‘problem’ features outlined in Table 2. Genetic data has long been used for species delimitation in this group (e.g. [86], [87]) thus standardization and expanding these activities via DNA barcoding is a natural progression. DNA barcoding is also being used to enhance understanding of species limits in seed plants, either via contributing towards the discovery of cryptic species or serving as an independent arbiter between competing taxonomies (e.g. [8], [59], [88]).

Many professions involve making or using plant identifications (e.g. taxonomists, ecologists, conservationists, foresters, agriculturalists, forensic scientists, customs and quarantine officers [89]). In terms of using DNA barcoding for plant identification, it is of course necessary to match the question at hand with the discriminatory power of the technique. As discussed in Section 2, there are many situations where the current barcoding approach will result in identification to ‘species group’ rather than species. However, for some applications, even a DNA barcode with relatively modest discriminatory power can be useful. Obvious situations include (1) geographically focused studies aiming to distinguishing among the diversity at a given site or region, where many of the samples are not necessarily closely related, and particularly where juvenile material and plant fragments require identifications; (2) species in trade, where the challenge is often to distinguish between a set of target species, and often distantly related potential substitutes or to identify members of higher taxonomic groups (e.g. family, genus) rather than particular species; and (3) where the identification problem relates to unfamiliarity with a given species such that the user may have no idea even what family a given species belongs to. In this situation, identification to a group of related species is useful as it can narrow down the total range of possible alternatives and also enable targeted use of morphological keys or expert consultation to obtain a final identification where required. This ‘species group identification’, followed by subsequent ‘tie-breaker’ analyses is particularly likely to be useful in species-rich systems where there is a shortage of available taxonomic expertise.

Table 3 lists some of the studies to-date using DNA barcoding *sensu lato* as a plant identification tool. One class of applications is ecological forensics, where DNA barcoding is used to identify plant roots, seedlings, or cryptic life stages (e.g. fern gametophytes). DNA barcoding offers a practical route to obtaining identifications in these situations [19], [90], [91]. Kesanakurthi et al. [90] used *rbcL* sequences alone to make assignments to species (or species groups) for 85% of all root samples examined, permitting a detailed examination of the ecological factors that contributed to the subterranean spatial organization of plant diversity in an old-field community. Likewise, DNA barcoding can provide identifications where material has been processed in one way or another, such as analyzing the diet of herbivores [45], [92]–[94], food products (e.g. [95]), or the components of herbal medicines (e.g. [96]). For instance, Baker and Little (Table 3) used *matK* DNA barcodes to highlight misidentified plant species in herbal supplements. Over a quarter of the commercially available herbal supplements of Black Cohosh they tested did not contain the target north American species *Actea raceomosa*, and instead contained Asian species of *Actea* as substitutes. Another emerging application of DNA barcoding in plants is the identification of protected species in trade. There are about 29000 plant species protected by CITES (Convention on International Trade in Endangered Species of Wild Fauna and Flora; http://www.cites.org/eng/disc/species.shtml), and developing effective methods to distinguish CITES-listed from non-CITES listed species is important. Ogden et al. [97] developed a SNP genotyping approach based on *matK* DNA barcodes to distinguish between traded timber products of Ramin (*Gonostylus*) species which are CITES protected, and other con-familial species or anatomically similar but distantly related species, which are not. A potential impediment for the use of the DNA barcoding in these and other regulatory frameworks is that species assignments may lack the definitiveness required in a court of law, more acutely in plants than in animals, but true for all organisms. Nonetheless, as a tool for initial identification, DNA barcoding may prove invaluable in this context even with its current limitations. In many circumstances identification to a larger taxonomic group is all that is required and this can be done in a definitive manner: e.g. for most of the plant species listed on CITES it is an entire genus or family that is listed, rather than individual species (e.g. cycads, orchids, Cactaceae, *Euphorbia*).

**Table 3:** Applications of DNA barcoding in plants.

Application	Barcode markers used	Notes	Ref.
Identification of cryptic orchid species	*matK*		[8]
Identification of cryptic *Conocephalum* (bryophyte) spp.	*rbc*L		[85]
Identification of cryptic *Herbertus* (bryophyte) species	*rbcL*+*matK*+*trnH-psbA*+nrITS	*matK* and nrITS rates as the best performing regions	Bell unpub.
Identification of cryptic *Taxus* species	nrITS, *trnL-F*, *trnH-psbA*, *matK*, *rbcL*	nrITS, *trnL-F* rated as the best performing regions	[59]
Identification of seedlings in tropical forest plots	nrITS, *trnH-psbA*, *rbcL*, *matK* (and other regions)	*trnH-psbA* was rated as the best performing region	[19]
Community phylogenetics of a tropical forest plot	*rbcL+trnH-psbA+matK*		[20], [21]
Identification of Chinese medicinal plants: Polygonaceae	*trnH-psbA*		[106]
Identification of Chinese medicinal plants: Fabaceae	nrITS2, *matK*		[56], [107]
Identification of *Phyllanthus* species in herbal medicines	*trnH-psbA*		[96]
Identification of *Actaea* species in herbal supplements	*matK*		[108]
Identification of medicinal plant species	nrITS2, nrITS, *matK*, *rbcL*, *psbA-trnH* (and other regions)	nrITS2 rated as the best performing marker	[54]
Identification among berry species in foods	nrITS	*matK* and *trnH-psbA* did not show enough variation	[95]
Assessing the plant components of honey	*trnL* P6 loop		[109]
Identification of invasive species (*Cardamine*)	nrITS, *trnL* intron, *trnL-F*		[110]
Identification of invasive species (*Hydrocotyle*)	*trnH-psbA*	*matK* proved troublesome to amplify so was discarded	[111]
Molecular identification of roots in grassland communities	*rbcL*		[90]
Identification of *Osmunda* gametophytes	*rbcL*		[91]
Identification of an aquatic fern gametophyte	*rbcL*, *trnL-F*, *trnL intron* (and other regions)	*trnL intron* less informative due to low coverage in reference database	[112]
Identification of traded fern species	*rbcL* (and other regions)		[113]
Identification of CITES listed ramin timber and products	*matK* (SNP assay of barcode sequence)		[97]
Identification of poisonous plants	*matK*, *trnH-psbA* and other regions	*matK* preferred (along with 2 single copy nuclear regions)	[114]
Identification of plant components of herbivore diet	*trnL* P6 loop		[45]
Identification of plant components of herbivore diet	*trnL* intron		[92], [115]
Identification of plant components of herbivore diet	*trnH-psbA*		[94]
Proof-of-concept for application to forensics	*trnH-psbA*, *trnL-F*		[116]

The table includes studies using a range of barcoding markers (beyond the *rbcL*+*matK* core barcode) to capture the broad spectrum of current applications.

The establishment of an appropriate reference library is a critical pre-requisite for these and other applications of DNA barcoding. This requires the generation of DNA barcode data from well identified and vouchered samples. There are multiple geographically- and taxon-based projects underway contributing towards this reference library (Table 4). In addition to these projects, there is sequence information archived in GenBank. This is particularly extensive for some of the barcode markers (Table 1) and provides a useful resource for identifications. However, many of the existing GenBank sequences lack validation in the form of voucher information and links to other metadata, and database curation is largely left in the hands of individual users, making it difficult to detect and remove mis-identified specimens or contaminated sequences. In contrast samples adhering to the BARCODE standard in GenBank and databases such as the Barcode of Life Datasystems (BOLD, [98]) are much more robust: they contain links between vouchers, sequences, trace files and other metadata.

**Table 4:** Examples of plant DNA barcoding projects underway or in the planning stage in 2011.

Project	Lead Institute
TreeBOL: Barcoding the world's tree species	The New York Botanic Garden
GrassBOL: Barcoding grasses and grass-like plants	Adelaide University and University of British Columbia
Flora of the Kruger National Park	University of Johannesburg
Flora of the Area de Conservacion Guanacaste Costa Rica	University of Pennsylvania
Flora of Korea	Korea University
Plant Barcoding China: DNA barcoding of 5000 Chinese plant species	Kunming Institute of Botany
All-genera: DNA barcoding of representatives of all angiosperm genera	The New York Botanic Garden
DNA barcoding of Centre for Tropical Forestry Plots	Smithsonian Institute
DNA barcoding Chinese medicinal plants	Institute of Medicinal Plant Development Beijing
DNA barcoding the flora of Wales	National Botanic Garden of Wales
DNA barcoding British bryophytes	Royal Botanic Garden Edinburgh

## 4. Research, Tools and Technology Required to Support DNA Barcoding in Plants

To effectively scale plant DNA barcoding for widespread use, a supporting infrastructure is needed. The two areas requiring the most attention are the development of laboratory protocols and informatics support. In this section we provide an annotated ‘wish-list’ of immediate priorities.

### 4.1. Laboratory protocols

*Cost-effective storage protocols for plant tissue samples*: Silica gel desiccation is the most frequently used method of preserving plant material for DNA extraction. Once desiccated, there are a wide diversity of storage practices adopted by different laboratories ranging from room temperature to refrigeration to frozen tissue archives. Guidelines need developing as to the most cost effective long-term storage options for silica dried samples in different climates.

*Protocols and guidelines for DNA extraction and sequencing from herbarium specimens*:The world's herbaria represent an exceedingly rich resource of millions of plant samples. However, obtaining DNA sequences from herbarium specimens can be far from routine. Assessments are required of the efficacy of different extraction, PCR and sequencing protocols in relation to taxonomic group and specimen age. A project addressing this issue is underway as part of the Synthesys programme JRA4 (http://www.synthesys.info/II_JRA_4.htm).

*Continued improvement of PCR and sequencing protocols for regions rich in mononucleotide repeats*: New polymerases have improved sequence quality for regions containing mononucleotide repeats [36], [37]. Further experimentation and optimization are required to increase sequencing success, particularly from samples containing long mononucleotide repeats.

*Development of DNA barcoding primers and a system to record and predict which primers will work well in a given taxonomic group*: As discussed earlier, work is required on *matK* primers to increase the rate of recovery from land plants. An automated system to predict which primer set(s) will work for a given taxonomic group will greatly improve laboratory success rates.

*Development of robust multiplex PCR protocols*: The establishment of multiplex PCR reactions that can routinely amplify the core *rbcL*+*matK* barcode and supplementary markers simultaneously will greatly reduce laboratory costs and the potential for laboratory error.

*Enhancement of mini-barcodes for degraded DNAs*:The P6 loop of the *trnL* intron is a useful option for sequencing highly degraded DNAs [44]. The nrITS2 region is also short enough in many plant groups to amplify with partially degraded samples [54]. Development of mini-barcode primers from barcoding regions such as *rbcL* and *matK* are required to expand this toolkit [99].

*An empirical review of the extent of paralogy and polymorphism problems for nrITS*: As discussed previously, it is important to obtain a quantitative review of the extent and phylogenetic distribution of situations in which nrITS is problematic versus situations in which it can serve as a useful component of a plant DNA barcode.

As outlined in Sections 1.2c and 2 above, there is also a general need for continued exploration of opportunities to utilize emerging sequence data and new technologies to enable routine and cost-effective access to nuclear sequence data, and to generally improve the efficiency of sequencing large sample sets.

### 4.2. Informatics support tools for data management and analysis

The management and analysis of DNA barcoding data in plants carries additional challenges beyond those relating to the use of a single marker (*CO1*) for animal barcoding. Firstly, the plant barcode involves managing and analyzing more data per sample: it involves a core-barcode of two markers and the potential use of supplementary markers. Secondly, due to different degrees of user effort and/or recovery success it is inevitable that the global plant barcoding database will contain a set of samples which have variable coverage of core and supplementary barcoding markers, leading to challenges of analyzing or interrogating partially overlapping datasets. Thirdly, most of the supplementary markers will be non-coding and often unalignable outside of a given genus – necessitating the development of additional routines for data management and analysis. Finally, incongruent signals from barcode markers sequenced from the same set of individuals is likely, given the propensity of plants to hybridize and the different modes of inheritance for the markers (uniparental versus biparental). Incongruence may also result from processing additional markers – the potential for laboratory mixups increases with each added step. Data management and data analysis tools that need integrating into a user-friendly workflow to facilitate high-throughput plant barcoding include:

#### *a. Data management tools*

*Tools to check for incongruence within markers to detect chimeric assemblies:* Low efficiency PCR combined with low-level contamination or mistaken assembly of forward and reverse sequences from different samples can lead to chimeric sequences. These can be difficult to identify in contigs with poor sequence overlap which can occur when sequencing markers with frequent read termination by mononucleotide repeats. Tools are required to automatically query segments of individual sequences to check for different affinities.

*Tools to deconvolute sequencing chromatograms with low quality values:* Although new polymerases have improved sequence quality for regions containing mononucleotide repeats [36], [37], the polymerases can still be stymied by long repeats. Software for reprocessing of sequencing chromatograms to deconvolute the peaks in over-laid traces and then output individual trace files from which quality scores can be calculated would be useful.

*Tools to check for contaminants/sample mix-ups:* In plant barcoding projects aiming to sequence multiple markers from large sample sets there is the possibility of sample mix-ups or contaminants leading to the wrong sequence being attributed to a given sample. While this may be ameliorated by workflow checks (e.g. production of parallel DNA extracts from individual samples), tools are required to efficiently check if the closest matching sequence in the database is sensible. This involves (i) establishing whether the closest matching sequence is from the same genus/family etc. and (ii) establishing whether there are individuals in the database that ought to be the closest match but are not.

*Tools to check for plant barcode sequence anomalies (e.g. ‘sequence feature checkers’):* Tools are required to automatically check plant barcoding sequences for editing errors or pseudogenes. For coding sequences, variants that cause stop-codons or frameshifts are detectable via translation of DNA sequences to amino acids. Amino acid composition can further be used to flag unusual sequences for more extensive verification. Another practical problem in plant barcoding projects is the inadvertent inclusion of reverse complemented sequences in data analyses (this is not uncommon for non-coding markers where analysis does not involve multiple sequence alignments). This problem can be detected via a workflow that uses highly conserved motifs to orient sequences and/or detection of discrepancies in sample affinities in comparisons between pairwise alignment-based distance algorithms that use only one sequence orientation, with those that calculate the minimum distance between each pair of sequences no matter their orientation (e.g. BLAST). Abnormal secondary structure and base composition for nrITS could also be diagnostic of potential problems.

#### *b. Data analysis tools*

*Analytical tools to implement analyses based on partial recovery of barcoding markers:* During the establishment of a plant barcoding reference library, there will be many unsampled taxa and varying depth of sample coverage for some markers, but not others. It will be necessary to develop efficient pipelines which (i) allow users to select sets of samples that have directly comparable coverage for a given set of markers, and (ii) to additionally be able to invoke analyses which effectively combine samples with partial and complete coverage of a set of barcode markers and still give meaningful signal in the presence of missing data.

*User friendly tools for implementing species discrimination analyses based on unaligned data:* Non-coding regions are widely used in addition to the *rbcL*+*matK* core-barcode, but some of these markers can be difficult or impossible to align in sample sets containing species from different genera. User friendly tools are required for (a) implementing species discrimination and identification routines that use pairwise global alignments or are ‘alignment-free’ (e.g. [11], [100], [101]), and, (b) automated selection of data partitions of alignable groups of samples for a given marker, and the subsequent production of stepped alignment blocks (e.g. [20], [21]).

*Tools for detecting microinversions:* Microinversions are not uncommon in non-coding regions and may lead to erroneous groupings of samples [31]. Tools are required to automatically identify and where necessary correct them, so that they can be accounted for in analyses.

Although individual solutions are available for some of the challenges outlined above, there is a general need for the integration of a range of analytical routines into a single easy-to-use work-flow to provide comparable informatics support for multi-marker barcoding in plants, along the lines of the available informatics support for *CO1* barcoding in animals.

## 5. Concluding Remarks

Much of this paper has focused on spelling out the challenges and difficulties for plant barcoding. Some of these challenges are non-trivial. In particular it is clear that the discrimination success of plant barcodes is lower than that found in many animal groups such as fishes, birds and butterflies. Despite these challenges, plant DNA barcodes will prove extremely useful for numerous applications such as ecological forensics, identification of traded materials, undertaking identifications where there is a shortage of taxonomic expertise available, and assisting species discovery in some plant groups. Future technological advances will undoubtedly lead to improvements over current approaches, but the key step is assembling large DNA sample sets representing the earth's botanical diversity, supported by voucher specimens, and indexed via DNA sequences. This will provide the framework for current applications, and future developments, in the coordinated use of DNA sequence data to tell plant species apart.

## Supporting Information

Table S1 **Discrimination success from 42 plant barcoding studies using plastid markers or nrITS.**
(DOC)
