# Supplementary material for: Choosing and Using a Plant DNA Barcode
Source: PLoS One. 2011 May 26;6(5):e19254. doi: 10.1371/journal.pone.0019254 (PMC3102656; doi:10.1371/journal.pone.0019254)
Supplement: Table S1 — Discrimination success from 42 plant barcoding studies using plastid markers or nrITS. (DOC) [file pone.0019254.s001.doc]

**Table S1: Discrimination success from 42 plant barcoding studies using plastid markers or nrITS.**

| **Study group** | **Sample strategy** | **Highest discrimination success (plastid markers)** | **Discrimination success (nrITS)** | **Notes** | **Ref.** |
| --- | --- | --- | --- | --- | --- |
| Floristic: Canadian flora | 251 individuals, 92 species, 32 genera | 65-70% (various combinations of plastid loci) |  |  | [1] |
| Floristic: Canadian local flora | 513 individuals, 436 species, 269 genera (ca 70% of local flora) | 97% (*rbcL*+*matK*+*trnH-psbA*+*rpoC1*+*atpF-atpH*) |  | *rbcL*+*matK =* 93%, *rbcL*+*matK*+*trnH-psbA =* 95% | [2] |
| Floristic: diet analyses | Environmental DNAs from faecal samples queried against local reference databases and GenBank | ca 50% (*trnL* P6 loop) |  |  | [3] |
| Floristic: Italian trees | 156 individuals, 50 species, 24 genera | 73% (*trnH-psbA*) |  |  | [4] |
| Floristic: medicinal plants | nrITS2 6685 individuals, 4800 species, 753 genera; *trnH-psbA* 2018 individuals, 1433 species, 551 genera (GenBank sequences) | 73% (*trnH-psbA*) | 93% (nrITS2) |  | [5] |
| Floristic: Neotropical trees | up to 368 individuals, 223 species, 125 genera | < 70% (*trnH-psbA*) | Up to 80% (nrITS) but based on 41% sequencing success. | Adding other plastid markers made no difference to discriminatory power | [6] |
| Floristic: Neotropical trees | 1035 individuals, 296 species, 181 genera | >98% (*rbcL*+*matK*+*trnH-psbA*) |  |  | [7] |
| Floristic: Neotropical trees | 288 individuals, 143 species, 108 genera | 93% (*rbcL*+*matK*+*trnH-psbA*) |  | *rbcL*+m*atK* = 89%, *trnH-psbA*+*matK* = 93% | [8] |
| Floristic: South Africa and Costa Rica | 172 individuals, 86 species, 57 genera | 91% (*matK*) |  | Adding other plastid markers made no difference to discriminatory power | [9,10] |
| Floristic: species pairs | 96 individuals, 96 species, 48 genera | 88% (*trnH-psbA* + either *rbcL*, *rpoC1* or rpoB) | 82% for nrITS1 (but based on 60% sequencing success) |  | [11] |
| Floristic: various | 259 individuals, 95 species, 34 genera | 70-75% (various combinations of plastid loci) |  | A larger data set (907 samples from 445 angiosperm, 38 gymnosperm and 67 cryptogam species) also showed broadly equivalent performance of multiple plastid markers combinations | [12] |
| Floristic: various | Genbank sequences of 50,790 plant nrITS2 sequences |  | nrITS2: ca 74-76% for angiosperms, 67% for gymnosperms, 88% for ferns, 77% for mosses |  | [13] |
| Taxon (clade) based: bryophytes from NE China | 80 individuals, 36 species, 34 genera | 90% (*rbcL*) | 66% (nrITS2) | Adding other plastid markers made no difference to discriminatory power | [14] |
| Taxon (clade) based: pteridophytes of Japan | 597 individuals, 597 species, 60 genera | 80% ( *trnH-psbA* + *rbcL*) |  |  | [15] |
| Taxon (clade) based: pteridophytes of NW Europe | 77 individuals, 52 taxa, 50 species (*rbcL*), 74 individuals, 47 taxa, 45 species (*trnL-*F) | 100% (rbcL+trnL-F) |  | Polyploid species are fused with maternal diploid progenitors in the estimation of discrimination success (e.g. lower value would be obtained if recent polyploids were treated as separate species). The *rbcL* fragment was 1300 bp, rather than the usual barcoding region. | [16] |
| Taxon based: *Acacia* | 56 individuals, 4 species | 100% (*matK*) |  |  | [17] |
| Taxon based: African Podostemaceae | 23 individuals, 11 species, 6 genera | 100% (*matK*) |  |  | [18] |
| Taxon based: *Agalinis* | (92 samples, 29 species [26 species in some analyses as some species are treated as synonyms] | ca 67% (*trnL*-*trnF*) |  | Values are % samples correctly assigned rather than species discriminated. Several plastid marker combinations gave similar discriminatory power; using an alternative taxonomy, discrimination success was higher (>90%) | [19] |
| Taxon based: *Alnus* | 131 individuals, 26 species | 64% (*trnH-psbA*) | 77% (nrITS) |  | [20] |
| Taxon based: Asteraceae | 3940 individuals, 2315 species, 494 genera from Genbank |  | 76% (nrITS2) | An additional data set 110 individuals, 63 species, 48 genera gave >90% discrimination for *matK* and also  *trnH-psbA* | [21] |
| Taxon based: *Araucaria* | 42 individuals, 17 species | 32% (various marker combinations produced similar results) |  | Adding other plastid markers made no difference to discriminatory power | [22] |
| Taxon based: *Asterella* s.l. | 98 individuals, 39 species | 90% (*rbcL*) |  | Adding other plastid markers made no difference to discriminatory power | [22] |
| Taxon based: *Berberis* | 58 individuals, 13 species | 23% (*matK*+*rbcL* or *matK*+*trnH-psbA* ) | 23% (nrITS) | Combining nrITS and plastid markers increases success to 31% | [23] |
| Taxon based: *Carex* | 93 individuals, 34 species | 57% (*matK*) | 25% (nrITS) | Adding other plastid markers made no difference to discriminatory power | [24] |
| Taxon based: *Carex* and *Kobresia* from Canadian Arctic | 109 individuals, 26 species, 2 genera | 95% (*matK*) |  | Adding one of several other plastid markers increases resolution to 100% | [25] |
| Taxon based: Caryoteae (Palms) | 39 individuals, 27 species, 4 genera | 81% (*rbcL*+*matK*+*trnH-psbA*) | 92% (nrITS2) |  | [26] |
| Taxon based: *Crocus* | 131 individuals, 86 species | 92% (*matK*, *trnH-psbA*, *ndhF, rps8-rpl36*) |  |  | [27] |
| Taxon based: cycads | 96 individuals, 74 taxa, 11 genera |  | 90% (nrITS) |  | [28] |
| Taxon based: cycads | 69 individuals, 69 species, 11 genera (three target genera + outgroups) | e.g. 79% (*Dioon -*  *atpF-atpH*+*psbK-psbI*); 52% (*Ceratozamia* - *atpF-atpH*, *psbK-psbI*, *rpoc1*, *matK*); 67% *(Zamia*  *atpF-atpH*, *psbK-psbI*, *rpoc1)* |  | In *Ceratozamia,* the combination of *atpF-atpH*, *psbK-psbI*, *rpoc1*, *matK,* nrITS2 led to 78% success; in *Zamia*  *atpF-atpH*, *psbK-psbI*, *rpoc1*, adding nrITS2 led to 75% success | [29] |
| Taxon based: Euphorbiaceae | Taxon-based 1183 samples, 871 species, 66 genera from genbank |  | 97% (nrITS); 91% (nrITS2) | 50 individuals from 42 species were also used to test *rbcL* and *matK*; discrimination success is ca 90% | [30] |
| Taxon based: Fabaceae | 1355 individuals, 1079 species, 409 genera | 80% (*matK*) |  |  | [31] |
| Taxon based: *Ficus* | 33 individuals, 11 species | 64% (*trnH-psbA*) | 100% (nrITS) |  | [23] |
| Taxon based: *Gossypium* | 51 individuals, 4 species | 25% (*trnH-psbA*) | 100% (nrITS) |  | [23] |
| Taxon based: Grimmiaceae | 73 individuals, 31 species, 4 genera | 71% (*trnH-psbA+rps4*) |  |  | [32] |
| Taxon based: *Inga* | 44 individuals 26 species | <69% (various 3-4 marker barcodes) |  |  | [22] |
| Taxon based: Lemnaceae | 97 individuals, 31 species, 6 genera | 93% (*atpF-atpH*) |  | Values are % samples correctly assigned rather than species discriminated. 14/19 species with N>1 individuals resolved as monophyletic (74%). Adding other plastid markers made no improvement | [33] |
| Taxon based: Meliaceae | 34 individuals, 17-19 species, 2 genera | 13% (*psbB-psbT-psbN*) | 67% (nrITS) |  | [34] |
| Taxon based: Myristicaceae (*Compsoneura*) | 40 individuals, 8 species | 95% (*matK*+*trnH-psbA*) |  | Values are % samples correctly assigned rather than species discriminated | [35] |
| Taxon based: *Picea* | 132 individuals, 33 species | 25% (*rbcL*+*matK* or *matK*+*psbK-psbI*) |  | Various other combinations of 3 marker barcodes gave equivalent results; using 6 markers gives 29% successful discrimination | [36] |
| Taxon based: *Quercus* | 30 individuals, 12 species | 0% (various markers) |  |  | [4] |
| Taxon based: *Solanum* | 104 individuals of 73 species | 12% (*trnH-psbA*) | 41% (nrITS) |  | [37] |
| Taxon based: *Taxus* | 47 individuals, 11 lineages from 8 named taxa | 100% (*trnL*-F) | 100% (nrITS2 = 45%) |  | [38] |

The intention of the table is to summarize the resolving power of plastid and nrITS barcodes (rather than to compare among different plastid markers). Thus the table lists the best performing plastid markers for a given study, rather than listing the performance of every marker tested (different studies have trialed different sets of markers and primers). No attempt has been made to standardize among the different methods used for species discrimination success. The reference numbers in the table refer to those in the self contained bibliography appended below.

**References cited in Table S1**

1. Fazekas AJ, Burgess KS, Kesanakurti PR, Graham SW, Newmaster SG, et al. (2008) Multiple multilocus DNA barcodes from the plastid genome discriminate plant species equally well. PLoS ONE 3: e2802.

2. Burgess KS, Fazekas AJ, Kesanakurti PR, Graham SW, Husband BC, et al. (2011) Discriminating plant species in a local temperate flora using the *rbcL*+*matK* DNA barcode. Methods in Ecology and Evolution DOI: 10.1111/j.2041-210X.2011.00092.x.

3. Valentini A, Miquel C, Nawaz MA, Bellemain EVA, Coissac E, et al. (2009) New perspectives in diet analysis based on DNA barcoding and parallel pyrosequencing: the *trnL* approach. Molecular Ecology Resources 9: 51-60.

4. Piredda R, Simeone MC, Attimonelli M, Bellarosa R, Schirone B (2010) Prospects of barcoding the Italian wild dendroflora: oaks reveal severe limitations to tracking species identity. Molecular Ecology Resources 11: 72-83.

5. Chen S, Yao H, Han J, Liu C, Song J, et al. (2010) Validation of the ITS2 region as a novel DNA barcode for identifying medicinal plant species. PLoS ONE 5: e8613.

6. Gonzalez MA, Baraloto C, Engel J, Mori SA, Pétronelli P, et al. (2009) Identification of Amazonian trees with DNA barcodes. PLoS ONE 4: e7483.

7. Kress WJ, Erickson DL, Jones FA, Swenson NG, Perez R, et al. (2009) Plant DNA barcodes and a community phylogeny of a tropical forest dynamics plot in Panama. Proceedings of the National Academy of Sciences 106: 18621-18626.

8. Kress WJ, Erickson DL, Swenson NG, Thompson J, Uriarte M, et al. (2010) Advances in the use of DNA barcodes to build a community phylogeny for tropical trees in a Puerto Rican forest dynamics plot. PLoS ONE 5: e15409.

9. Lahaye R, Savolainen V, Duthoit S, Maurin O, van der Bank M (2008) A test of *psbK-psbI* and *atpF-atpH* as potential plant DNA barcodes using the flora of the Kruger National Park (South Africa) as a model system. Available from Nature Precedings <http://hdlhandlenet/10101/npre200818961>

10. Lahaye R, van der Bank M, Bogarin D, Warner J, Pupulin F, et al. (2008) DNA barcoding the floras of biodiversity hotspots. Proceedings of the National Academy of Sciences 105: 2923-2928.

11. Kress WJ, Erickson DL (2007) A two-locus global DNA barcode for land plants: The coding *rbcL* gene complements the non-coding *trnH-psbA* apacer region. PLoS ONE 2: e508.

12. CBOL-Plant-Working-Group (2009) A DNA barcode for land plants. Proceedings of the National Academy of Sciences 106: 12794-12797.

13. Yao H, Song J, Liu C, Luo K, Han J, et al. (2010) Use of ITS2 region as the universal DNA barcode for plants and animals. PLoS ONE 5: e13102.

14. Liu Y, Yan H-F, Cao T, Ge X-J (2010) Evaluation of 10 plant barcodes in Bryophyta (Mosses). Journal of Systematics and Evolution 48: 36-46.

15. Ebihara A, Nitta JH, Ito M (2010) Molecular species identification with rich floristic sampling: DNA barcoding the pteridophyte flora of Japan. PLoS ONE 5: e15136.

16. de Groot GA, During HJ, Maas JW, Schneider H, Vogel JC, et al. (2011) Use of *rbcL* and *trnL-F* as a two-locus DNA barcode for identification of NW-European ferns: an ecological perspective. PLoS ONE 6: e16371.

17. Newmaster SG, Ragupathy S (2009) Testing plant barcoding in a sister species complex of pantropical *Acacia* (Mimosoideae, Fabaceae). Molecular Ecology Resources 9: 172-180.

18. Kelly LJ, Ameka GK, Chase MW (2010) DNA barcoding of African Podostemaceae (river-weeds): A test of proposed barcode regions. Taxon 59: 251-260.

19. Pettengill JB, Neel MC (2010) An evaluation of candidate plant DNA barcodes and assignment methods in diagnosing 29 species in the genus *Agalinis* (Orobanchaceae). Am J Bot 97: 1391-1406.

20. Ren B-Q, Xiang X-G, Chen Z-D (2010) Species identification of *Alnus* (Betulaceae) using nrDNA and cpDNA genetic markers. Molecular Ecology Resources 10: 594-605.

21. Gao T, Yao H, Song J, Zhu Y, Liu C, et al. (2010) Evaluating the feasibility of using candidate DNA barcodes in discriminating species of the large Asteraceae family. BMC Evolutionary Biology 10: 324.

22. Hollingsworth ML, Clark A, Forrest LL, Richardson JR, Pennington RT, et al. (2009) Selecting barcoding loci for plants: evaluation of seven candidate loci with species-level sampling in three divergent groups of land plants. Molecular Ecology Resources 9: 439-457.

23. Roy S, Tyagi A, Shukla V, Kumar A, Singh UM, et al. (2010) Universal plant DNA barcode loci may not work in complex groups: A case study with Indian *Berberis* species. PLoS ONE 5: e13674.

24. Starr JR, Naczi RFC, Chouinard BN (2009) Plant DNA barcodes and species resolution in sedges (*Carex*, Cyperaceae). Molecular Ecology Resources 9: 151-163.

25. Le Clerc-Blain J, Starr JR, Bull RD, Saarela JM (2010) A regional approach to plant DNA barcoding provides high species resolution of sedges (*Carex* and *Kobresia*, Cyperaceae) in the Canadian Arctic Archipelago. Molecular Ecology Resources 10: 69-91.

26. Jeanson ML, Labat J-N, Little DP (2011) DNA barcoding: a new tool for palm taxonomists? Annals of Botany in press.

27. Seberg O, Petersen G (2009) How many loci does it take to DNA barcode a crocus? PLoS ONE 4: e4598.

28. Sass C, Little DP, Stevenson DW, Specht CD (2007) DNA barcoding in the Cycadales: Testing the potential of proposed barcoding markers for species identification of cycads. PLoS ONE 2: e1154.

29. Nicolalde-Morejón F, Vergara-Silva F, González-Astorga J, Stevenson DW, Vovides AP, et al. (2010) A character-based approach in the Mexican cycads supports diverse multigene combinations for DNA barcoding. Cladistics 26: 1-15.

30. Pang X, Song J, Zhu Y, Xie C, Chen S (2010) Using DNA barcoding to identify species within Euphorbiaceae. Planta Med 76: 1784-1786.

31. Gao T, Sun Z, Yao H, Song J, Zhu Y, et al. (2011) Identification of Fabaceae plants using the DNA barcode *matK*. Planta Medica 77: 92-94.

32. Liu Y, Cao T, Ge X-J (2011) A case study of DNA barcoding in Chinese Grimmiaceae and a moss recorded in China for the first time. Taxon 60: 185-193.

33. Wang W, Wu Y, Yan Y, Ermakova M, Kerstetter R, et al. (2010) DNA barcoding of the Lemnaceae, a family of aquatic monocots. BMC Plant Biology 10: 205.

34. Muellner AN, Schaefer H, Lahaye R (2011) Evaluation of candidate DNA barcoding loci for economically important timber species of the mahogany family (Meliaceae). Molecular Ecology Resources 10.1111/j.1755-0998.2011.02984.x.

35. Newmaster SG, Fazekas AJ, Steeves RAD, Janovec J (2008) Testing candidate plant barcode regions in the Myristicaceae. Molecular Ecology Notes 8: 480-490.

36. Ran J-H, Wang P-P, Zhao H-J, Wang X-Q (2010) A Test of seven candidate barcode regions from the plastome in *Picea* (Pinaceae). Journal of Integrative Plant Biology 52: 1109-1126.

37. Spooner DM (2009) DNA barcoding will frequently fail in complicated groups: An example in wild potatoes. American Journal of Botany 96: 1177-1189.

38. Liu JIE, Möller M, Gao L-M, Zhang D-Q, Li D-Z (2010) DNA barcoding for the discrimination of Eurasian yews (*Taxus* L., Taxaceae) and the discovery of cryptic species. Molecular Ecology Resources 11: 89-100.
